# Litter Inputs Promoted Soil Organic Carbon Formation by Increasing Particulate Organic Carbon on the Eastern Edge of the Tibetan Plateau

**DOI:** 10.3390/plants15111645

**Published:** 2026-05-27

**Authors:** Zhanqing Ma, Weidi Zhou, Man Su, Leisong Bai, Weijia Han, Yanjie Gu, Chenglong Han

**Affiliations:** 1 Minhe Hui and Tu Autonomous County Ecological Environment Monitoring Station, Haidong 810800, China; mazhq2013@163.com; 2 State Key Laboratory of Plateau Ecology and Agriculture, Qinghai University, Xining 810016, China; zhouweidi010206@163.com (W.Z.); suman895637@163.com (M.S.); baileisong1291@163.com (L.B.); hanweijia30@163.com (W.H.); 3 College of Agriculture and Animal Husbandry, Qinghai University, Xining 810016, China; guyanjie@126.com

**Keywords:** litter input, litter variety, soil labile organic carbon fractions, carbon decomposition

## Abstract

Forest soil organic carbon (SOC) accumulation is regulated by plant-derived carbon inputs and soil environmental conditions, but the relative roles of litter composition and soil physicochemical properties in regulating SOC fractions remain unclear in high-elevation forest ecosystems. This study aimed to determine whether variation in SOC among different forest types was mainly associated with particulate organic carbon (POC) or mineral-associated organic carbon (MAOC), and to identify the relative roles of litter characteristics, soil physicochemical properties, microbial biomass, and enzyme activities in regulating SOC fractions. Four forest types on the eastern edge of the Tibetan Plateau were investigated: broadleaved poplar forest (PLF), larch forest (LXF), seabuckthorn forest (SBF), and Dasiphora shrubland (DS). PLF had the highest SOC and POC contents (75.8 and 48.5 g kg^−1^, respectively), whereas MAOC did not differ significantly among forest types. SOC was strongly positively correlated with POC (R^2^ = 0.74, *p* < 0.001), but not with MAOC, indicating that SOC variation was mainly associated with POC accumulation. PLF litter contained higher labile and recalcitrant carbon components, including soluble sugar (19.9 g kg^−1^), starch (30.1 g kg^−1^), lignin (94.6 g kg^−1^), and litter carbon (404 g kg^−1^). Partial least squares path modeling showed that soil physicochemical properties had the strongest direct path relationship with SOC variation (*p* < 0.001), while litter composition was positively associated with microbial biomass and POC formation (*p* < 0.01). These findings suggest that POC formation was the main fraction-level feature associated with SOC accumulation, while soil properties and litter composition were related to SOC through different pathways.

## 1. Introduction

In the context of global climate change, forest ecosystems serve as critical terrestrial carbon sinks, mitigating rising atmospheric CO_2_ concentrations [1,2,3,4]. Within these ecosystems, soil represents one of the largest organic carbon reservoirs, playing a key role not only in regional carbon balance but also in maintaining soil fertility and ecosystem stability [5,6,7]. Soil organic carbon (SOC) has historically been treated as a homogeneous pool [8]. However, growing evidence indicates that SOC consists of distinct components—each characterized by different stability levels, turnover rates, formation mechanisms, and environmental sensitivities [9,10]. Among these, particulate organic carbon (POC) and mineral-associated organic carbon (MAOC) are recognized as key functional fractions that reflect soil carbon activity and long-term stability [11,12]. Understanding whether POC or MAOC predominantly drives SOC dynamics in a given ecosystem is therefore essential for accurately assessing forest carbon sequestration potential and guiding science-based forest management strategies.

The eastern Qinghai–Tibet Plateau features rugged terrain and a cool, humid climate, representing a typical high-altitude forest and shrubland region in China [13,14]. Ecologically sensitive, this area serves as a key indicator of regional and global carbon cycle dynamics [15]. With ongoing afforestation and natural secondary succession, diverse forest types have emerged in the region [16]. These forest types vary significantly in species composition [17], canopy structure [18], litter input [19], and litter chemical properties [20]. These differences further lead to notable variations in soil moisture and temperature regimes, physicochemical properties, and microbial community characteristics [21,22], which may drive distinct patterns in SOC composition. Although previous studies have examined the spatial distribution of SOC in the forests of the eastern Qinghai–Tibet Plateau [11,23], the relative contributions of POC and MAOC across different forest types, as well as the underlying drivers, remain systematically unquantified.

From a process-based perspective, litter input serves as the principal source of SOC, with its quantity and quality directly shaping the characteristics of carbon entering the soil [24]. Soil physicochemical properties and nutrient availability indirectly regulate SOC accumulation by affecting the preservation and transformation efficiency of organic matter [25]. Meanwhile, microorganisms and their extracellular enzymes play a central role in decomposing litter and modifying carbon substrates [26]. These interconnected processes govern the allocation of carbon into microbial biomass, POC, and MAOC, thereby influencing the overall stability of the soil carbon pool [8]. However, in environmentally distinct and forest-type-rich regions such as the eastern Qinghai–Tibet Plateau, there remains a scarcity of quantitative evidence clarifying how litter characteristics, soil physicochemical properties, and microbial activity jointly drive the formation and persistence of POC and MAOC.

Although previous studies have examined SOC stocks, soil properties, microbial activity, and litter decomposition in forest ecosystems of the eastern Qinghai–Tibetan Plateau [13,14,24], most of them have focused on total SOC or individual environmental factors. The relative contributions of POC and MAOC to SOC variation across contrasting forest types remain insufficiently quantified [9,10,11,12]. Moreover, few studies have simultaneously evaluated how litter chemical composition, soil physicochemical properties, microbial biomass, and extracellular enzyme activities are associated with the formation of POC and MAOC in this high-elevation ecotone [8,13,19,23]. Therefore, a more integrated analysis is needed to clarify whether SOC variation among forest types is mainly associated with labile particulate carbon accumulation or mineral-associated carbon stabilization.

The eastern edge of the Tibetan Plateau provides a suitable region for addressing this gap because it is characterized by complex topography, high-elevation climatic conditions, ecological sensitivity, and diverse forest and shrubland types formed through afforestation and natural succession [13,14,15,16]. The four forest types selected in this study-broadleaved poplar forest, larch forest, seabuckthorn forest, and Dasiphora shrubland-differ in vegetation composition, stand development, litter quality, and soil conditions [16,19,20]. These contrasts provide a useful natural gradient for examining forest-type differences in SOC fractions and for identifying the relative importance of litter-derived substrates, soil physicochemical properties, and microbial processes [8,13,19,23]. Compared with previous studies that mainly focused on total SOC or single-factor controls, this study integrates SOC fractionation, litter chemistry, soil properties, microbial biomass, and enzyme activity to provide new evidence for the factors associated with SOC accumulation in high-elevation forest ecosystems.

Based on this, the present study focuses on four typical forest types in the eastern Qinghai–Tibet Plateau—broadleaved Poplar forest, Larix forest, Seabuckthorn forest, and Dasiphora shrubbery—collecting soil samples from the 0–20 cm depth. We systematically quantified the contents of SOC, POC, and MAOC, together with key litter carbon components, soil physicochemical properties, microbial biomass, and extracellular enzyme activities related to carbon, nitrogen, and phosphorus acquisition. We hypothesized that: (1) SOC fractions would differ among forest types, with POC showing stronger variation than MAOC because of its higher sensitivity to vegetation-derived carbon inputs and soil environmental conditions; (2) variation in total SOC would be more closely associated with POC accumulation than with MAOC, indicating that the more active particulate carbon pool contributes substantially to forest-type differences in SOC; and (3) litter characteristics and soil physicochemical properties would be associated with SOC accumulation through different pathways, with litter traits mainly linked to microbial biomass and POC formation, whereas soil physicochemical properties would show a stronger direct relationship with SOC variation. Based on these hypotheses, this study aims to: (1) compare the variation in SOC and its components (POC and MAOC) across different forest types; (2) clarify the relative contributions of litter characteristics, soil physicochemical properties, and microbial factors to POC and MAOC; and (3) identify the key pathways associated with SOC accumulation. The results are expected to provide mechanistic insights into soil carbon sequestration processes in forest ecosystems of the eastern Qinghai–Tibet Plateau and to offer theoretical support for regional forest management and the enhancement of carbon sink functions.

## 2. Results

### 2.1. SOC Content and Its Fractions

**Figure 1:**
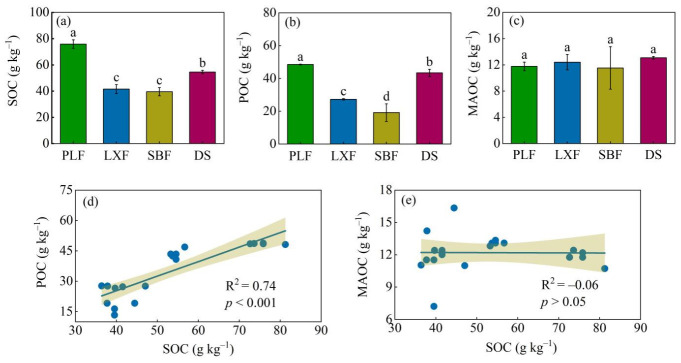
Contents of soil organic carbon (SOC) (**a**), particulate organic carbon (POC) (**b**), mineral-associated organic carbon (MAOC) (**c**), and relationships between SOC and POC or MAOC (**d**,**e**) across different forest types. Bars represent mean ± SE (n = 5). Different lowercase letters indicate significant differences among forest types (*p* < 0.05).

The SOC content differed significantly among forest types (Figure 1a). Among the four forest types studied, PLF showed the highest SOC content, which was significantly greater (*p* < 0.05) than that in the other three forest types. DS ranked second, while LXF and SBF contained lower and similar SOC levels. These results suggest that PLF has the strongest capacity for SOC accumulation in this region. When examining carbon fractions, the variation in POC closely mirrored that of SOC (Figure 1b). PLF and DS contained higher POC contents, with PLF being significantly higher than the other forest types (*p* < 0.05). SBF had the lowest POC content, and LXF exhibited an intermediate level. In contrast, no significant differences were observed in MAOC across the four forest types (Figure 1c), with MAOC remaining relatively stable regardless of forest type.

Correlation analysis further supported these patterns. A strong positive correlation was detected between SOC and POC (R^2^ = 0.74, *p* < 0.001; Figure 1d), indicating that forest types with higher POC also accumulated more SOC. However, no significant correlation was found between SOC and MAOC (Figure 1e), suggesting that MAOC did not drive the observed variation in SOC across forest types. In conclusion, the increase in SOC observed in this study was primarily attributable to the accumulation of POC, rather than changes in MAOC. This highlights that in these forest ecosystems, the more labile, faster-cycling POC pool plays a dominant role in governing total SOC storage.

### 2.2. Soil and Litter Properties

Significant differences in litter chemical composition were observed among forest types (Table 1), providing key context for the variation in SOC components. In terms of carbon constituents, PLF exhibited the highest overall carbon input: soluble sugar and starch contents reached 19.9 g kg^−1^ and 30.1 g kg^−1^, respectively, both significantly greater (*p* < 0.05) than in the other three forest types. This indicates that PLF litter supplies the most abundant pool of readily decomposable carbon. PLF also contained the highest levels of cellulose, lignin, and total phenols, reflecting a substantial reserve of more recalcitrant carbon. In contrast, the DS litter had a significantly lower total carbon content, while the LXF and SBF forests showed intermediate levels. Regarding litter nutrients, SBF had the highest nitrogen content, resulting in the lowest C/N ratio (16.3), whereas PLF and LXF displayed higher C/N ratios, with PLF reaching 30.6. This suggests that PLF litter is relatively carbon-rich and nitrogen-poor.

Soil physicochemical properties also varied markedly across forest types (Table 1). Soil pH in PLF averaged approximately 6.9, similar to DS and higher than in LXF and SBF. SWC was higher in PLF and SBF and lower in LXF. BD value was lowest in PLF, indicating a more porous soil structure, while SBF had the highest BD, reflecting a relatively compacted soil. PLF contained the highest levels of AN and TN among all forest types. DS had a relatively high TN but a lower AN compared to PLF. AP was highest in SBF, with PLF at an intermediate level. TP was slightly elevated in PLF and DS, and lowest in SBF.

### 2.3. Soil Microbial Biomass and Activity

**Figure 2:**
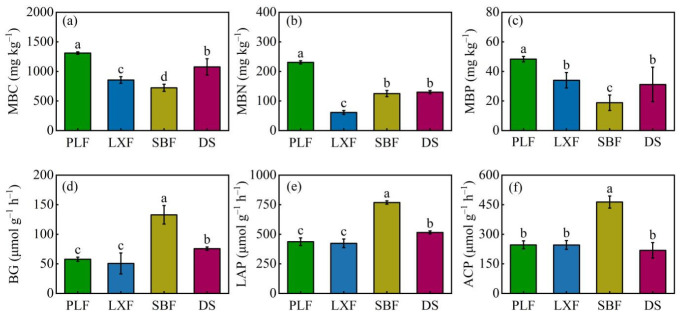
(**a**–**f**) Soil microbial biomass carbon, nitrogen, and phosphorus (MBC, MBN, and MBP) and soil extracellular enzyme activities, including β-1,4-glucosidase (BG), leucine aminopeptidase (LAP), and acid phosphatase (ACP), across different forest types. Bars represent mean ± SE (n = 5). Different lowercase letters indicate significant differences among forest types (*p* < 0.05).

Microbial biomass exhibited clear differentiation among the four forest types (Figure 2a–c). MBC in PLF was significantly higher (*p* < 0.05) than in the other forest types. Similarly, MBN was much greater in PLF, while LXF showed the lowest MBN. MBP was also highest in PLF, whereas SBF had notably lower MBP. These results indicate that PLF supports not only a larger microbial population but also greater reserves of carbon, nitrogen, and phosphorus within the microbial biomass.

In contrast, extracellular enzyme activities related to carbon, nitrogen, and phosphorus acquisition displayed a different pattern (Figure 2d–f). BG activity, associated with carbon acquisition, was significantly higher in SBF (*p* < 0.05) compared to the other forests, while PLF and LXF showed relatively lower activities. Similarly, LAP activity, involved in organic nitrogen decomposition, was highest in SBF, followed by DS, with PLF and LXF exhibiting lower levels. ACP activity was also greatest in SBF, while no significant differences were observed among the other three forest types.

### 2.4. Relationship Between SOC, POC, MAOC, and Soil and Litter Properties

**Figure 3:**
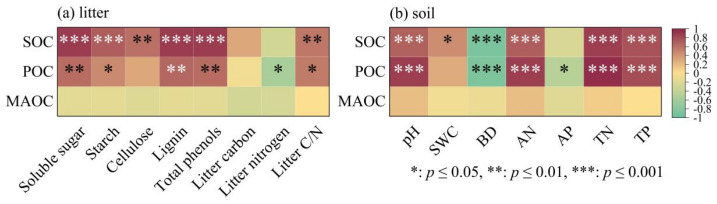
Correlation analysis between carbon fractions and soil litter properties.

Correlation analysis further clarified the relationships among carbon components, litter properties, and soil physicochemical variables (Figure 3). From the litter perspective (Figure 3a), both SOC and POC showed significant positive correlations with most litter carbon constituents. SOC was strongly correlated (*p* < 0.05) with soluble sugars, starch, cellulose, lignin, and total phenols. POC exhibited a similar pattern, displaying positive correlations with soluble sugars, starch, lignin, and total phenols, along with a significant negative correlation with litter nitrogen content and a positive correlation with litter C/N ratio. In contrast, MAOC showed only weak or non-significant relationships with litter characteristics.

From the perspective of soil physicochemical properties (Figure 3b), SOC and POC responded very similarly to soil environmental factors. Both were positively correlated with soil pH and nutrient levels (AN, TN, and TP) and negatively correlated with BD. Conversely, MAOC displayed much weaker correlations with these soil factors and did not follow the same response patterns as SOC and POC.

**Figure 4:**
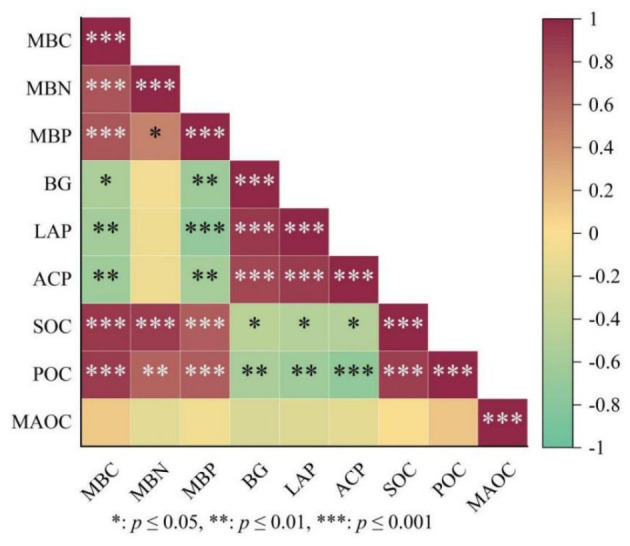
Correlation analysis between microbial properties and soil carbon fractions, enzyme activity.

Correlation analysis further revealed distinct relationships between microbial indicators and SOC components (Figure 4). MBC, MBN, and MBP were strongly intercorrelated, with coefficients close to 1 and high significance (*p* < 0.001), indicating tight coupling among microbial nutrient pools. All three microbial biomass measures showed significant positive correlations with SOC and POC, whereas their associations with MAOC were relatively weak. This suggests that higher microbial biomass corresponds to greater SOC and POC accumulation, but does not strongly influence MAOC levels. In contrast, extracellular enzyme activities—BG, LAP, and ACP—were also highly correlated with each other, reflecting coordinated acquisition of carbon, nitrogen, and phosphorus in these soils. Notably, the activities of all three enzymes were significantly negatively correlated with SOC and POC, with LAP and ACP showing particularly strong inverse relationships.

**Figure 5:**
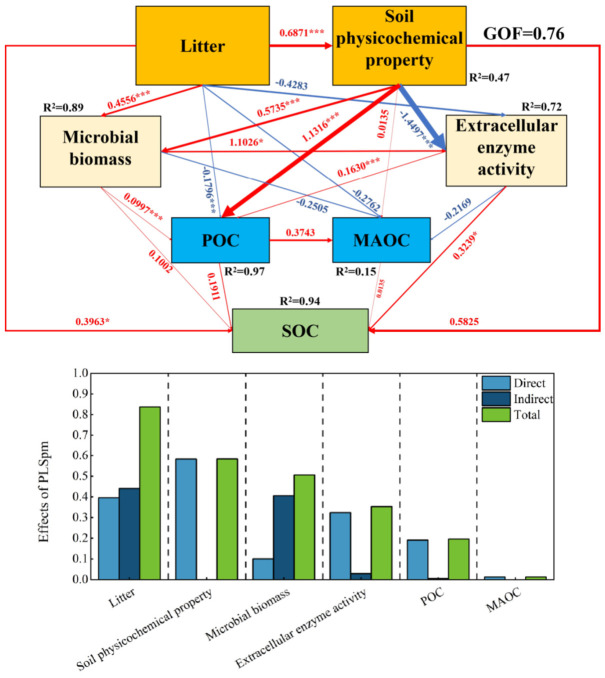
Partial least squares path model (PLS-PM) illustrating the relationships among litter characteristics, soil physicochemical properties, microbial factors, and SOC fractions. Red and blue arrows indicate negative and positive path relationships, respectively. Numbers above lines indicate standardized path coefficients. R^2^ values beside the latent variables denote coefficients of determination. *, *p* < 0.05; ***, *p* < 0.001.

The direct and indirect relationships among litter characteristics, soil physicochemical properties, microbial factors, and SOC components, as derived from partial least squares path modeling (Figure 5). The PLS-PM results indicated that soil physicochemical properties and litter characteristics were strongly associated with SOC variation. Soil physicochemical properties showed the strongest positive path relationship with SOC, while litter characteristics were positively associated with microbial biomass and POC accumulation. Soil physicochemical properties were positively related to microbial biomass but negatively associated with extracellular enzyme activity.

## 3. Discussion

### 3.1. Forest-Type Differences in Litter Characteristics and SOC Fractions

Our results demonstrate that the quantity of litter inputs significantly influences the formation of SOC from litter-derived carbon. Among the forest types, PLF exhibited the greatest variation in SOC fractions—POC and MAOC—in response to litter addition. The increase in SOC storage observed in this study is likely attributable to elevated carbon input resulting from higher litter productivity in PLF [23]. Previous studies have highlighted the impact of litter input on SOC storage [27], with litter quality and quantity being key determinants of SOC stabilization [11,23]. We found that the rise in SOC content was primarily driven by POC accumulation, rather than by changes in MAOC (Figure 1). This contrasts with classical theoretical frameworks that emphasize the dominant role of MAOC in long-term soil organic matter (SOM) accumulation and stabilization [28].

Soil physicochemical properties played a critical role in mediating POC accumulation. BD, pH, AN, and TN displayed distinct dynamics across the four forest types, likely due to interactions between soil characteristics [29] and microbial metabolism [30]. Lower BD enhances soil porosity, improving water retention, gas exchange, and habitat conditions that favor POC accumulation [31]. Moreover, higher porosity associated with lower BD can stimulate microbial activity [32,33], regulating organic matter decomposition and promoting POC formation. A neutral to slightly alkaline pH generally supports microbial growth [34]. Elevated nitrogen availability (both AN and TN) likely enhances microbial activity and the production of metabolic byproducts derived from repeated litter inputs, further facilitating the transformation of litter carbon into POC [35,36,37].

The relatively stable MAOC content observed across forest types in this study contrasts with previous findings, where greater litter inputs led to increased MAOC accumulation [38]. MAOC formation typically relies on the adsorption of organic matter onto mineral surfaces and slow microbial processing [11,39]. In our system, however, microbial necromass—a potential precursor to MAOC—may have undergone rapid recycling within the active microbial loop rather than being stabilized into the mineral-associated fraction. This suggests that the substantial SOC accumulation in PLF was driven primarily by POC buildup, without a corresponding increase in MAOC.

### 3.2. Effects of Litter Quality on Soil SOC Accumulation

While traditional ecological theory posits that low-quality, slowly decomposing litter promotes the accumulation of plant-derived compounds in SOC [40], our findings reveal a more nuanced relationship between litter chemistry and SOC dynamics. In PLF, litter contained abundant labile carbon (soluble sugars and starch) alongside high levels of recalcitrant compounds (lignin, cellulose, and total phenols) (Table 1), suggesting that litter quality exerts a multifaceted control over the chemical composition of SOC [41].

Our observations support a contemporary microbial-mediated framework for SOC accumulation, which emphasizes the central role of microbial growth and activity. This model proposes that microbes contribute to SOC mainly through the production of necromass derived from labile litter components, thereby linking high-quality litter to greater SOC accumulation via enhanced microbial growth [42,43]. However, we also found that recalcitrant litter constituents (lignin, cellulose, and phenols) played a significant role in soil carbon accumulation [44]. The higher POC content in PLF—where litter quality was both high in labile and recalcitrant fractions—likely reflects differential decomposition rates driven by litter chemistry [45], rather than immediate microbial utilization. When labile substrates are abundant, microbes preferentially metabolize them [38], allowing less-decomposed, recalcitrant materials to accumulate as POC. Our results align partly with this expectation, showing greater POC accumulation under combined high- and low-quality litter inputs in PLF compared to other forest types. Collectively, these patterns suggest that SOC stabilization results from litter material passing through a “microbial filter” [38,42,43], where microbial processing regulates the fate of carbon into various SOC pools.

Furthermore, the higher litter C:N ratio in PLF compared to SBF and DS points to early microbial immobilization of carbon during decomposition. This process can make partially decomposed litter an important nitrogen source, while microbes continue to exploit labile carbon from newly added litter [46,47,48]. The negative correlations between extracellular enzyme activities and SOC/POC suggest a possible trade-off between enzyme-mediated organic matter decomposition and carbon retention [13,42,43]. Higher BG, LAP, and ACP activities may indicate stronger microbial investment in acquiring C, N, and P from organic substrates under relatively limited resource conditions [13,46,47,48]. Such enhanced enzyme activity could be associated with faster decomposition of particulate organic matter, thereby reducing POC retention and limiting SOC accumulation [42,43,49]. In contrast, PLF showed higher SOC and POC contents but relatively lower extracellular enzyme activities, which may be related to its greater litter-derived carbon supply and more favorable soil nutrient conditions. Under these conditions, microbes may rely more on readily available substrates and invest less in extracellular enzyme production [13,50]. Therefore, the inverse relationships between enzyme activities and SOC/POC may reflect differences in microbial resource-acquisition strategies among forest types rather than a direct inhibitory effect of enzymes on SOC accumulation. The input of substrate-rich litter (high in soluble sugars and starch) in our experiment likely supplied ample labile carbon, which may explain the lower extracellular enzyme activities observed in PLF (Figure 3) [50]. In other words, even when enzyme activity directed toward plant substrates is reduced under high-quality litter conditions, slower turnover of decomposing litter could still lead to proportionally greater retention of litter-derived carbon over time. Such interactions between fresh and older litter are likely common in managed forest ecosystems through natural litterfall and residue inputs.

Compared with other forest ecosystems in the eastern Qinghai–Tibetan Plateau and similar subalpine regions, the forests studied here showed comparable weakly acidic to neutral soil pH, relatively high soil water content in broadleaved and shrub-dominated forests, and clear forest-type differences in soil nitrogen availability and litter C/N ratio [13,14,16]. These similarities indicate that the coupling among litter characteristics, soil physicochemical properties, and SOC fractions observed in this study may also be relevant to other high-elevation forest ecosystems with comparable climatic and vegetation conditions [31,40]. However, the magnitude of SOC and POC accumulation may vary among regions because of differences in stand age, soil parent material, elevation, forest structure, and decomposition environment [14,19,31]. Therefore, extrapolation of the present findings to other forests should consider the comparability of soil and litter properties, particularly those listed in Table 1.

Although aboveground litter was the main focus of this study, other carbon sources and transformation pathways may also contribute substantially to SOC formation. Fine root production and turnover represent important belowground carbon inputs in forest soils [16,41]. Previous studies in the eastern Qinghai–Tibetan Plateau have shown that fine root dynamics vary among forest types, suggesting that root-derived carbon inputs may also differ among vegetation types [16]. In addition, soil invertebrates can regulate litter fragmentation, microbial colonization, and the transfer of plant-derived carbon into soil organic matter [37,44]. Their effects on litter decomposition may depend on litter species, season, and litter chemical composition [40,44]. Therefore, the higher POC accumulation observed in PLF may reflect not only aboveground litter characteristics, but also unmeasured belowground root inputs and decomposer-mediated processes.

Litter turnover rates may also differ among vegetation types in this region. Differences in litter C/N ratio, lignin, cellulose, soluble carbon compounds, and phenolic compounds can influence decomposition rate and carbon retention [40,44]. In the eastern Qinghai–Tibetan Plateau, low temperature, seasonal moisture variation, and differences in forest structure may further regulate microbial and faunal decomposition processes [13,16]. However, seasonal litterfall, litter decomposition rate, fine root turnover, root exudation, and soil invertebrate activity were not directly quantified in this study. Future studies should combine repeated litterfall collection, litterbag decomposition experiments, fine-root production measurements, and soil fauna observations or exclusion experiments to better resolve the pathways of SOC formation across forest types.

This study was based on a single sampling campaign conducted in 2024 and should therefore be interpreted as a single-season, cross-sectional comparison of SOC fractions, litter characteristics, soil physicochemical properties, microbial biomass, and enzyme activity among forest types. The present dataset does not allow us to evaluate whether the observed relationships remain stable under interannual climatic variability. This limitation is particularly important in high-elevation ecosystems, where temperature, precipitation, soil moisture, and seasonal freeze–thaw processes may strongly influence litter decomposition, microbial activity, enzyme production, and SOC dynamics. Therefore, the relationships identified in this study should be interpreted as associations observed during the sampling period rather than as temporally stable mechanisms of SOC accumulation.

In addition, humic substance fractionation was not conducted in this study. Although POC and MAOC fractionation allowed us to distinguish between particulate and mineral-associated SOC pools, this physical fractionation approach does not fully characterize the humification degree, chemical transformation, or stability of soil organic matter [51]. Because humic acids, fulvic acids, and humin were not quantified, the transformation of plant-derived and microbial-derived organic matter into more stable humic forms could not be directly evaluated. As a result, the mechanistic interpretation of SOC stabilization in this study remains partly limited and should be restricted to the physical SOC fractions measured here. Future studies should combine multi-year monitoring of litterfall, litter decomposition, fine root turnover, soil microclimate, microbial activity, enzyme activity, and SOC fractions with humic substance fractionation to better assess the temporal stability, humification degree, chemical stability, and transformation pathways of soil organic matter across different forest types.

## 4. Materials and Methods

### 4.1. Study Sites

The study was conducted in Haidong City, Qinghai Province, China, situated on the eastern fringe of the Tibetan Plateau (102°26′ E, 35°45′ N), at the transitional zone between the Tibetan Plateau and the Loess Plateau. The region features complex topography and a semi-arid climate, with a mean annual temperature of 7.9 °C, annual precipitation ranging from 350 to 400 mm, and an elevation of approximately 2600 m. Soils in this area exhibit notable spatial heterogeneity, shaped by both climatic and geographic factors. Forest types are diverse, encompassing typical broadleaf forests, coniferous forests, and shrublands [52]. Through ongoing afforestation and natural secondary succession, a variety of forest stand types have developed, each characterized by distinct species composition, canopy structure, and litter input dynamics.

### 4.2. Experimental Design and Sampling

In August 2024, coinciding with the peak period of aboveground productivity, we sampled four forest sites along a recovery chronosequence. Three of these sites originated from artificial planting and underwent subsequent natural growth. The broadleaved poplar forest (88 years, PLF) was dominated by *Populus cathayana* Rehder. The larch forest (54 years, LXF) was dominated by *Larix gmelinii* var. *principis-rupprechtii* (Mayr) Pilger. The seabuckthorn forest (30 years, SBF) was dominated by *Hippophae rhamnoides* subsp. *sinensis* Rousi. The fourth site, dasiphora shrubbery (DS), was established naturally after cropland conversion 15 years prior. For each forest type, one 20 m × 20 m plot was established. Within each plot, five 1 m × 1 m subplots were randomly selected as subplot-level replicates. In each subplot, aboveground litter was first collected from a 30 cm × 30 cm area to determine litter biomass and chemical properties. After litter collection, the remaining surface litter was removed, and soil samples were collected from the 0–20 cm layer within the same 1 m × 1 m subplot using a 4 cm diameter soil auger. Five soil cores were randomly collected within each subplot and composited into one subplot-level soil sample. Therefore, each forest type contained five subplot-level replicates, and litter and soil samples were spatially paired at the subplot scale. Litter samples were oven-dried at 60 °C to constant weight for biomass determination.

### 4.3. Soil and Litter Physicochemical Properties Analysis

Soil organic carbon (SOC) content was determined using the Walkley–Black dichromate oxidation method, in which organic carbon is oxidized by potassium dichromate under acidic conditions and then calculated according to the amount of oxidant consumed. Total nitrogen (TN) was measured by micro–Kjeldahl digestion, in which organic nitrogen is converted into ammonium and then quantified by distillation. Total phosphorus (TP) was analyzed after perchloric acid digestion using the molybdate–ascorbic acid colorimetric method. Available nitrogen (AN) was quantified using an auto–flow injection analyzer (AutoAnalyzer AA3, SEAL Analytical GmbH, Norderstedt, Germany), and available phosphorus (AP) was extracted using the Olsen method and then measured colorimetrically [53]. POC and MAOC were separated by sodium hexametaphosphate dispersion and particle–size fractionation, and their carbon contents were then determined [49]. Soil water content (SWC) was determined gravimetrically after drying at 105 °C to constant weight, and bulk density (BD) was calculated from the oven-dry mass and core volume. Soil pH was measured in a 1:2.5 soil–water suspension using a calibrated pH meter (Seven Excellence S400 (Mettler-Toledo, Columbus, OH, USA)).

Litter lignin content was determined by acetyl bromide digestion followed by UV-absorbance measurement [54]. Cellulose content was quantified using the cold anthrone reagent colorimetric method by Ververis et al., [55]. Soluble sugars and starch were extracted and measured according to the enzymatic-colorimetric procedure described by Cong Dien et al. [56].

For quality control, reagent blanks and duplicate samples were included during the analyses. The analytical precision was checked using duplicate measurements, and the relative errors were within the acceptable limits of the corresponding standard methods.

### 4.4. Analysis of Soil Microbial Biomass and Enzymatic Activity

Soil microbial biomass carbon (MBC), nitrogen (MBN), and phosphorus (MBP) were determined using the chloroform fumigation-extraction method. Fresh soil samples were fumigated with ethanol-free chloroform in a sealed dark chamber at 25 °C for 24 h, and microbial biomass was calculated based on the difference in extractable C, N, and P between fumigated and non-fumigated soil samples. For soil enzyme activity assays, the hydrolytic enzymes β-1,4-glucosidase (BG), leucine aminopeptidase (LAP), and acid phosphatase (ACP) were measured as indicators of C, N, and P cycling, respectively. Enzyme activities were determined using a fluorescence-based microplate assay with 4-methylumbelliferone (MUB)-labeled substrates. Fluorescence readings were taken on a microplate reader (Varioskan LUX, ThermoFisher Scientific, Waltham, MA, USA) with the excitation at 365 nm and emission at 450 nm. Activities were expressed as µmol substrate converted per gram of dry soil per hour (µmol g^−1^ h^−1^).

### 4.5. Statistical Analyses

Statistical analyses were performed in R (version 4.0.3). Before statistical analysis, the normality of data distribution was assessed using the Shapiro–Wilk test. Variables that did not meet the normality assumption were transformed before parametric analyses. Differences in litter traits, soil physicochemical properties, microbial biomass, extracellular enzyme activities, and soil carbon components (SOC, POC, and MAOC) across forest types were tested using one-way analysis of variance (ANOVA). Pairwise relationships among these variables were examined using Pearson correlation analysis. To evaluate the direct and indirect drivers of soil carbon components, we applied partial least squares path modeling (PLS-pm) via the “plspm” package in R, incorporating litter characteristics, soil properties, microbial biomass, and enzyme activities as predictors. The significance threshold was set at *p* < 0.05 for all tests. Figures were prepared using Origin 9.2 (OriginLab, Northampton, MA, USA).

## 5. Conclusions

This study compared SOC fractions, litter characteristics, soil physicochemical properties, microbial biomass, and enzyme activity among four forest types on the eastern edge of the Tibetan Plateau. The results showed that SOC variation among forest types was mainly associated with POC rather than MAOC. PLF had higher SOC and POC contents, which were accompanied by higher litter carbon components and more favorable soil physicochemical conditions. These findings suggest that POC formation is an important fraction-level feature associated with SOC differences among the studied forest types.

Litter biomass and litter chemical composition were related to SOC and POC accumulation, but the role of litter quality should be interpreted cautiously. The present results do not provide direct evidence that low-quality litter alone promotes more efficient POC accumulation. Instead, POC accumulation appeared to be associated with the combined effects of labile carbon components, recalcitrant structural compounds, microbial resource-acquisition strategies, and soil environmental conditions.

Importantly, the conclusions of this study are limited by the single-season sampling design and the absence of humic substance fractionation. The results should therefore be regarded as a cross-sectional comparison within the sampling period rather than direct evidence for interannual stability or long-term SOC accumulation mechanisms. In addition, because humic acids, fulvic acids, and humin were not quantified, this study cannot fully evaluate the humification degree or chemical stabilization of soil organic matter. Broader extrapolation of the findings to long-term SOC stabilization mechanisms in high-elevation forest ecosystems should therefore be made cautiously.

## Figures and Tables

**Table 1:** Soil and litter properties across different forest types. Values are mean ± SE (n = 5). F and *p* values indicate the significance of one-way ANOVA. Different lowercase letters indicate significant differences among forest types (*p* < 0.05).

	Parameters	PLF	LXF	SBF	DS	F	*p*
Litter	Litter biomass (g m^−2^)	36.5 ± 1.09 c	607 ± 18.2 b	8.40 ± 0.12 c	1316 ± 58.4 a	404	<0.001
	Soluble sugar (g kg^−1^)	19.9 ± 0.61 a	6.33 ± 0.09 c	7.59 ± 0.11 b	6.75 ± 0.08 bc	427	<0.001
	Starch (g kg^−1^)	30.1 ± 0.42 a	23.3 ± 0.20 b	17.5 ± 0.08 c	16.6 ± 0.05 d	704	<0.001
	Cellulose (g kg^−1^)	22.6 ± 0.30 a	16.8 ± 0.08 b	14.9 ± 0.09 c	11.3 ± 0.24 d	557	<0.001
	Lignin (g kg^−1^)	94.6 ± 1.83 a	63.3 ± 1.19 b	59.6 ± 0.71 b	60.9 ± 1.30 b	161	<0.001
	Total phenols (g kg^−1^)	21.9 ± 0.06 a	1.99 ± 0.15 d	4.81 ± 0.18 b	3.44 ± 0.04 c	5660	<0.001
	Litter carbon (g kg^−1^)	404 ± 1.50 a	342 ± 5.27 c	354 ± 1.60 b	243 ± 1.00 d	538	<0.001
	Litter nitrogen (g kg^−1^)	14.5 ± 0.08 b	11.7 ± 0.18 d	21.5 ± 0.06 a	12.7 ± 0.06 c	1603	<0.001
	Litter C/N	30.6 ± 0.75 a	29.2 ± 0.75 a	16.3 ± 0.22 c	22.0 ± 0.09 b	151	<0.001
Soil	pH	6.91 ± 0.00 b	6.53 ± 0.00 d	6.58 ± 0.02 c	7.03 ± 0.01 a	542	<0.001
	SWC (%)	43.9 ± 0.42 a	23.5 ± 0.66 c	43.6 ± 1.64 a	39.7 ± 0.89 b	91.3	<0.001
	Bulk density (g cm^−3^)	0.68 ± 0.00 c	0.89 ± 0.02 b	1.05 ± 0.02 a	0.85 ± 0.00 b	105	<0.001
	Available nitrogen (mg kg^−1^)	13.4 ± 1.18 a	9.76 ± 0.52 b	3.39 ± 0.25 c	12.1 ± 0.37 a	42.7	<0.001
	Available phosphorus (mg kg^−1^)	4.76 ± 0.24 b	1.89 ± 0.21 c	10.86 ± 0.74 a	3.68 ± 0.78 b	48.4	<0.001
	Total nitrogen (g kg^−1^)	5.60 ± 0.01 a	3.76 ± 0.12 c	3.53 ± 0.10 c	5.32 ± 0.02 b	172	<0.001
	Total phosphorus (g kg^−1^)	2.69 ± 0.02 a	2.47 ± 0.02 b	2.14 ± 0.04 c	2.40 ± 0.03 b	57.1	<0.001

## Data Availability

Restrictions apply to the datasets. The datasets presented in this article are not readily available because they are part of an ongoing study and further analyses are being conducted. Requests to access the datasets should be directed to hanchenglong2008@126.com.
